# Multivariate Associations Between the Geriatric Trauma Outcome Score (GTOS) and Injury Characteristics in the Geriatric Trauma Population

**DOI:** 10.7759/cureus.110472

**Published:** 2026-06-08

**Authors:** Vladimir Rubinshteyn, Vincent Giordano, Sandra Liju, Tara Harrington, Johnathon LeBaron, Paul S Issack

**Affiliations:** 1 Trauma Services, Richmond University Medical Center, New York, USA; 2 Trauma, American University of Antigua, St. John's, ATG; 3 Emergency Medicine, Richmond University Medical Center, New York, USA; 4 Orthopedic Surgery, Richmond University Medical Center, New York, USA

**Keywords:** anova, firth logistic regression models for mortality, geriatric trauma, geriatric trauma outcome score, hospitalized patients, icu patients, injury severity score (iss), linear regression analysis, older adult, outcome measure

## Abstract

Background

Trauma in geriatric populations represents a growing public health concern associated with elevated morbidity and mortality. The Geriatric Trauma Outcome Score (GTOS) is a widely used trauma severity metric developed to estimate clinical risk and in-hospital mortality among elderly trauma patients. It incorporates patient age, Injury Severity Score (ISS), and packed red blood cell (PRBC) transfusion status within 24 hours of admission. Although GTOS has shown clinical utility in geriatric trauma populations, limited literature has characterized the demographic and injury-related factors associated with elevated GTOS values in hospitalized patients.

Methods

This retrospective cohort study included patients aged ≥65 years admitted to a Level 1 trauma center in New York City between November 2024 and December 2025. Study variables encompassed demographics, body mass index (BMI), anticoagulation status, Glasgow Coma Score (GCS), mechanism of injury, and primary injury diagnosis. Descriptive statistics, univariate analyses, quartile stratification, and multivariable linear regression modeling were performed to identify factors associated with elevated GTOS values. Exploratory mortality analysis was conducted using the Firth penalized logistic regression, given the low frequency of mortality events.

Results

A total of 687 patients were included in the final analytic cohort. The mean patient age was 79.3 ± 8.4 years, and mean GTOS was 92.2 ± 15.6. Traumatic brain injuries (TBIs) and head injuries together represented the most prevalent diagnostic category (36.2%, n=249). In multivariable linear regression analysis, lower BMI, anticoagulation use, motor vehicle collisions, and selected injury diagnoses were significantly associated with GTOS values. Exploratory Firth penalized logistic regression demonstrated significant associations between mortality and both GTOS (OR 1.04, 95% CI 1.02-1.07, p=0.003) and GCS (OR 0.77, 95% CI 0.62-0.98, p=0.036).

Conclusion

Several demographic and injury-related factors, including lower BMI, anticoagulation use, motor vehicle collisions, and selected injury diagnoses, were significantly associated with GTOS values among hospitalized geriatric trauma patients. Higher GTOS values and lower GCS scores were each independently associated with mortality in exploratory analysis. These findings further characterize the clinical profile of geriatric trauma patients with elevated GTOS and support the continued utility of this score in clinical management settings.

## Introduction

Advances in medical care and preventive health measures have contributed to increased life expectancy across the general population. The number of adults aged 65 years and older in the United States has grown substantially over recent decades. According to the U.S. Department of Health and Human Services, the national population aged 65 and older rose from 37.2 million in 2006 to 49.2 million in 2016, and is projected to reach 98 million by 2060. In 2016, this age group represented 15.2% of the total population; that proportion is expected to grow to 21.7% by 2040 [1].

With the growth of this population, the number of geriatric patients presenting with traumatic injuries to emergency departments nationwide has also increased. A large analysis of Medicare inpatient insurance claims reported that geriatric trauma accounted for approximately 8.5% of all geriatric patient hospitalizations. Additionally, an analysis of data from the National Trauma Data Bank (NTDB) found that the proportion of geriatric trauma patients increased from 18% in 2005 to nearly 30% in 2015 [2,3]. Mortality rates following traumatic injury increase significantly with age, even when injury severity is similar. Outcomes in geriatric trauma patients are influenced by age-related changes in organ function, declining physiological performance, and the presence of numerous comorbidities and pre-existing conditions [4]. As the population of adults aged 65 years and older continues to grow, the incidence of geriatric trauma is also increasing. Tools that better gauge patient outcomes are important for guiding clinical management, informing treatment plans, supporting clinician decision-making, and facilitating goals-of-care discussions with patients and their families.

In this regard, several trauma scoring systems have been developed to estimate injury severity and predict related clinical outcomes. These trauma scores can be classified as either anatomical (i.e., Abbreviated Injury Scale (AIS) [5] and Injury Severity Score (ISS) [6]), physiological (i.e., Revised Trauma Score (RTS) [7,8] and Glasgow Coma Score (GCS) [9]), or combined scoring systems (i.e., Trauma and Injury Severity Score (TRISS) [10,11] and A Severity Characterization of Trauma (ASCOT) [12,13]).

ISS is one of the most widely used anatomical scoring systems. It is calculated by squaring and summing the AIS scores of the three most severely injured bodily regions (head, neck, face, thorax, abdomen, extremities, or external) and then computing an index ranging from one (minor) to 75 (fatal). An ISS score of ≥16 is indicative of major trauma. ISS is frequently used to "rank" injury severity, guide patient prognosis, and inform early triage decisions. Higher ISS values are linked to an increased need for intensive care, potential operations, and prolonged facility durations. As ISS is often correlated with the level of care intervention required, it is regularly used to guide interhospital transfer decisions, such as in determining whether patients may benefit from treatment at higher-level trauma centers with more advanced surgical equipment and radiological services. An extension of both RTS and ISS, TRISS is a widely used score which combines the ISS, the RTS, and a patient’s age to estimate the probability of survival following trauma [10].

The Geriatric Trauma Outcome Score (GTOS) was developed by Zhao, et al. in 2015 to predict in-hospital mortality among trauma patients aged 65 years and older using their age, ISS, and packed red blood cell (PRBC) transfusion status [14-16] as follows:

$GTOS = age + (2.5 * ISS) + 22 \space\text{(if given PRBCs)}$

The constant of 22 is added if a PRBC transfusion was given within 24 hours of hospital admission. This scoring system was later validated by the Prognostic Assessment of Life and Limitations After Trauma in the Elderly (PALLIATE) consortium and has been proposed as a tool to assist clinicians in facilitating goals-of-care discussions after traumatic injury. Additionally, Cook et al. provided a revised formulation, known as GTOS II, which was more effective than GTOS in predicting unfavorable discharge outcomes such as hospice care [17]. It is formulated as follows:

$GTOS = age + (0.71 * ISS) + 8.79 \space\text{(if given PRBCs)}$

Multiple studies have evaluated the predictive performance of the GTOS in the general geriatric trauma population. Analysis using the National Trauma Data Bank (NTDB) demonstrated that GTOS predicted mortality more accurately than its individual components alone [18]. Ravindranath et al. support this by arguing that GTOS has greater predictive capacity for mortality than age or ISS alone, with an area under the receiver operating characteristic curve (AUC) of 0.838 [19]. GTOS can also be used to predict both morbidity and mortality risk among hospitalized geriatric patients, with higher scores associated with increases in the number and types of in-hospital acquired complications and adverse outcomes. These scores can be used to identify high risk patients early and direct them to higher levels of care [20]. Arslan et al. reviewed 382 geriatric patients presenting to a trauma center with blunt force injuries and found that those with a GTOS of ≥95 had a higher probability of mortality at the 30th day post-trauma than their counterparts with a score <95 [21].

However, other studies suggest that the predictive power of GTOS for hospitalized patients may vary depending on patient populations and injury characteristics. The relationship between GTOS and non-fatal in-hospital acquired complications has not been well established [22]. Additionally, some studies have suggested that GTOS may underestimate mortality risk in patients with traumatic brain injuries (TBIs) and a GCS score ≤12 [23]. Critically ill geriatric patients are not well studied, which may limit clinicians' ability to extrapolate patient prognosis using ISS or GTOS to make decisions in settings such as the intensive care unit (ICU) [24]. In subgroups of patients with high predicted risk, GTOS tends to overestimate the risk of adverse outcomes [19]. In a study with geriatric ICU patients, Barea-Mendoza et al. reported aspects of both GTOS and TRISS to be deficient, with both scores overestimating mortality in higher-risk groups [25]. Furthermore, while advanced age, TBI, hypotension, and baseline preexisting conditions such as anticoagulation therapy are well established predictors of mortality in older trauma patients, GTOS does not include said comorbidities or biometric measures germane to the geriatric population in its calculation [26,27].

Additionally, Heppner et al. posit that existing comorbidities and functional limitations brought on by age should receive more attention as the number of geriatric patients in intensive care is expected to rise in the near future [28]. In terms of accuracy for mortality, one study analyzed geriatric patients who died within 30 days of treatment and derived a new score, the Geriatric Trauma Risk Indicator (GTRI). They found that compared to other scores like ISS, which had a specificity value of 7.5% when all compared scores (such as GTOS and ISS) were held at 90%, the GTRI had a much higher specificity value of 30.6%, indicating that more novel scoring systems may be better indicators of geriatric trauma patient outcomes [29]. Nevertheless, Huang et al. argue that GTOS still maintains high predictive accuracy and may even be used in settings outside of the geriatric population, such as the overall trauma patient pool [30].

Although GTOS has been extensively studied as a predictor of mortality and morbidity, relatively little literature has examined the relationship between GTOS and biometric or clinical covariates. As discussed previously, a shortcoming of the current GTOS formulation is its exclusion of pre-existing conditions. Therefore, understanding how these factors relate to differences in outcome scores remains an important research gap in the existing literature. As such, further evaluation of the relationship between GTOS and patient-specific and injury-related characteristics remains warranted. Accordingly, this study evaluated factors associated with elevated GTOS values among hospitalized geriatric trauma patients. Descriptive analyses, GTOS quartile stratification, univariate analyses, correlation analyses, and multivariable linear regression modeling were performed with GTOS treated as a continuous dependent variable. Exploratory mortality analysis was also performed using the Firth penalized logistic regression to account for low mortality event frequency and reduce potential overfitting.

## Materials and methods

Study design

This study was conducted as a retrospective cohort analysis of geriatric trauma patients admitted to Richmond University Medical Center, a Level 1 trauma center in New York City. The primary objective was to evaluate the relationship between key patient biometric, demographic, and injury-related factors and GTOS. The secondary objective was to evaluate the relationship between GTOS and in-hospital mortality. Data were obtained from the Richmond University Medical Center trauma registry, which prospectively collects standardized patient data for all trauma activations. The study period included all eligible patients presenting between November 1, 2024, and December 31, 2025. All identifying patient information was removed prior to data extraction, and each medical record was anonymized using randomly assigned study identification codes. 

Participants

All geriatric patients, or those patients aged 65 years or older, who underwent trauma activation at the Richmond University Medical Center emergency department and were subsequently admitted to a hospital service (i.e., Trauma Surgery, Orthopedics, etc.) were eligible for inclusion. Table 1 delineates the full inclusion criteria for the retrospective cohort while Table 2 outlines the study's exclusion criteria.

**Table 1:** Inclusion criteria of the retrospective cohort GTOS, Geriatric Trauma Outcome Score; ISS, Injury Severity Score; BMI, body mass index.

Inclusion criteria
Patients aged 65 years or older
Activated as a trauma in the emergency department
Admitted to one of the hospital’s services (i.e., Orthopedics)
Availability of complete data required to calculate GTOS, including age and ISS
Availability of complete triage data (i.e., BMI)

**Table 2:** Exclusion criteria of the retrospective cohort GTOS, Geriatric Trauma Outcome Score; ISS, Injury Severity Score.

Exclusion Criteria
Patients younger than 65 years
Discharge from the emergency department without admission, or departure against medical advice (AMA) without admission
Missing key variables required for GTOS calculation, including age or ISS
Duplicate patient entries
Patient declared dead on arrival in the emergency department without admission

Patients were excluded if they were younger than 65 years of age, discharged from the emergency department without admission, missing data required for GTOS calculation (including age and ISS), missing core triage data (i.e., BMI), had duplicate entries, or were declared dead on arrival. While all eligible patients in the retrospective cohort must satisfy all inclusion criteria, patients were excluded if they met at least one exclusion criterion. 

Variable selection

Patient-level variables were extracted from the trauma registry and organized into a fully anonymized and randomized dataset. Demographic and biometric variables included age, sex assigned at birth, body mass index (BMI), and anticoagulation use. Clinical and injury-related variables included mechanism of injury, GCS recorded in the emergency department, PRBC transfusion status within 24 hours of hospital admission, ISS, and primary injury diagnosis. Mechanisms of injury were categorized as falls, motor vehicle collisions, pedestrians struck (by motor vehicles or bicycles), assaults, bicycle accidents, and other or unknown mechanisms. Primary injury diagnosis was broken up into the following categories: TBIs, head injuries (including skull fractures and concussions), soft tissue injuries (including lacerations, abrasions, and contusions), long bone fractures, other fractures (including spinal and hip fractures), and other or unknown injuries (including unspecified dislocations and pneumothoraces). Within the analytical framework, TBIs and head injuries are grouped together as one injury diagnostic category. Post-admission and hospital course variables included hospital length of stay (LOS) recorded in calendar days and patient death recorded as a binary indicator variable. GTOS was calculated for each patient using the previously published formula described by Zhao et al. [14]: age + (2.5 × ISS) + 22 (if transfused within 24 hours of hospital admission).

Descriptive statistics were calculated to summarize baseline characteristics for the retrospective cohort. Continuous variables were summarized using means ± standard deviations (SDs) and discrete variables using medians with interquartile ranges (IQR) where appropriate. Categorical variables were summarized using frequencies and percentages in n (%) format. GTOS quartile analysis was performed to characterize successive clinical differences across increasing GTOS categories. Univariate analyses included Welch two-sample t-tests, one-way analysis of variance (ANOVA), Tukey post-hoc testing, and Spearman's *ρ* analysis. The primary multivariable analysis made use of Ordinary Least Squares (OLS) linear regression modeling with GTOS treated as a continuous dependent variable. Predictor covariates included sex, BMI, GCS, anticoagulation status, mechanism of injury, and primary injury diagnosis, with the latter two predictor variables using selected reference categories. Statistical significance was set as a two-tailed p-value of less than 0.05. All analyses were performed using Stata/BE version 17 (StataCorp LLC, College Station, TX, US) and R statistical software (R Foundation for Statistical Computing, Vienna, Austria). All scoring systems and indices utilized in this study (including GTOS, ISS, and GCS) are previously validated tools and were applied in accordance with their originally published methodologies. These are widely applied clinical indices and were employed for research purposes without modification to the original formulations.

Residual diagnostics and heteroskedasticity testing were subsequently performed. We tested for heteroskedasticity using a Breusch-Pagan test and applied heteroskedasticity-consistent robust standard errors (SEs) where necessary. Because overall mortality event frequency remained low (n=14), exploratory mortality analysis was undertaken using Firth penalized logistic regression to reduce small-sample bias and potential instability associated with conventional logistic regression.

This study was conducted using a retrospective review of fully anonymized and randomized patient data with no direct patient contact. The study protocol was submitted for institutional review board (IRB) review, and both a waiver of informed consent and Health Insurance Portability and Accountability Act (HIPAA) authorization were granted due to the retrospective design and use of anonymized data.

## Results

Descriptive statistics

A total of 1,936 geriatric trauma patients presenting to the Richmond University Medical Center Emergency Department were identified during the study period from November 1, 2024 to December 31, 2025. After applying inclusion and exclusion criteria, a total of 687 geriatric trauma patients were included in the final retrospective cohort.

The mean age was 79.3 ± 8.4 years, and 56.2% (n=386) were female patients, while 43.8% (n=301) were male patients. The mean BMI was 26.2 ± 6.0 kg/m². Injury severity was generally low, with a median ISS of four (IQR, 1-9). Most patients were recorded as alert and oriented upon initial presentation to the emergency department, with a median GCS of 15 (IQR, 15-15), creating an extreme leftward skew in the distribution (for context, the GCS range was 3-15). The mean GTOS was 92.2 ± 15.6. 

Falls were the predominant mechanism of injury, accounting for 89.7% (n=616) of the total injuries, with other mechanisms occurring less frequently. Anticoagulation therapy was present in 40.5% (n=278) of patients, and blood transfusion within 24 hours of hospital admission occurred in 2.0% (n=14). Primary injury diagnoses were distributed as follows: TBIs and head injuries together accounted for 36.2% (n=249) of injuries, soft tissue injuries were 28.5% (n=196), long bone fractures were 16.4% (n=113), other fractures were 7.6% (n=52), and other or unknown injuries were 11.2% (n=77). Median LOS was two days (IQR, 1-5) and mortality occurred in 2.0% (n=14) of patients.

Table 3 outlines the core demographic and biometric characteristics of the cohort while Table 4 details their corresponding injury and clinical summaries.

**Table 3:** Core demographic and biometric descriptive statistics of the study population SD, standard deviation; BMI, body mass index; kg/m², kilograms per square meter.

Variable	Value
Demographics	
Age (years), mean ± SD	79.3 ± 8.4
Female, n (%)	386 (56.2%)
Male, n (%)	301 (43.8%)
Biometrics	
BMI (kg/m²), mean ± SD	26.2 ± 6.0
Anticoagulation use, n (%)	278 (40.5%)

**Table 4:** Core injury and clinical descriptive statistics of the study population ISS, Injury Severity Score; IQR, interquartile range; GCS, Glasgow Coma Score; GTOS, Geriatric Trauma Outcome Score; SD, standard deviation; TBI, traumatic brain injury; LOS, length of stay.

Variable	Value
Injury severity	
ISS, median (IQR)	4 (1–9)
GCS, median (IQR)	15 (15–15) (range: 3–15)
GTOS, mean ± SD	92.2 ± 15.6
Mechanism of Injury	
Falls, n (%)	616 (89.7%)
Motor vehicle collisions, n (%)	15 (2.2%)
Pedestrians struck, n (%)	10 (1.5%)
Assaults, n (%)	10 (1.5%)
Bicycle accidents, n (%)	8 (1.2%)
Other or unknown mechanisms, n (%)	28 (4.1%)
Clinical factors	
Transfusion within 24h of admission, n (%)	14 (2.0%)
Primary diagnosis	
TBIs and head injuries, n (%)	249 (36.2%)
Soft tissue injuries, n (%)	196 (28.5%)
Long bone fractures, n (%)	113 (16.4%)
Other fractures, n (%)	52 (7.6%)
Other or unknown injuries, n (%)	77 (11.2%)
Hospital course	
LOS (days), median (IQR)	2 (1–5)
Mortality, n (%)	14 (2.0%)

**Figure 1:**
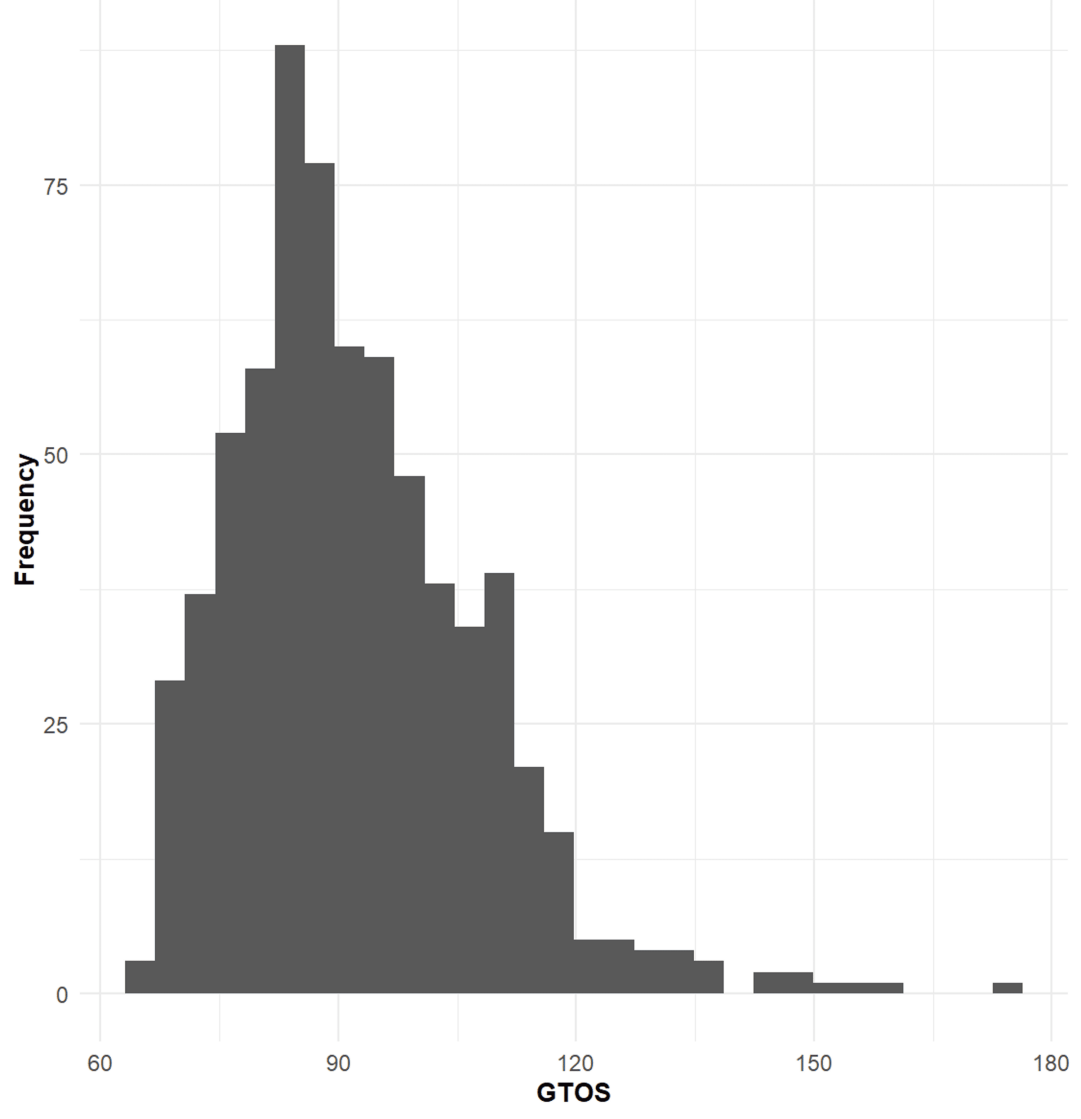
Distribution of the GTOS across the retrospective cohort GTOS, Geriatric Trauma Outcome Score.

The distribution of GTOS values across the retrospective cohort can be observed in Figure 1.

There is a noticeable rightward skew with a broad range of observed scores. Most patients clustered around moderate GTOS values. Several higher-score outliers were observed, reflecting patients with more severe overall injury burden and posing elevated clinical risk. 

GTOS quartile stratification analysis

Patients were subsequently stratified into GTOS quartiles to evaluate progressive demographic and clinical differences across increasing GTOS values. Mean GTOS values progressively increased from Q1 (75.0 ± 4.2) to Q4 (113.3 ± 12.0), as did anticoagulation use (Q1: 42 (24.4%) to Q4: 103 (60.2%)) and LOS (Q1: 1 (IQR, 1-2) to Q4: 4 (IQR, 2-8)). Mortality frequency increased non-significantly across quartiles. GCS remained relatively stable across quartiles because of the highly skewed distribution discussed earlier, though the results were similarly non-significant. The results are delineated in Table 5. 

**Table 5:** Clinical stratification by the GTOS quartile GTOS, Geriatric Trauma Outcome Score; SD, standard deviation; GCS, Glasgow Coma Score; IQR, interquartile range; BMI, body mass index; kg/m², kilograms per square meter; LOS, length of stay; ANOVA, analysis of variance.

Variable	Q1	Q2	Q3	Q4	Statistical test	Test statistic	p-value
n	172	172	172	171	—	—	—
GTOS, mean ± SD	75.0 ± 4.2	85.4 ± 2.5	95.1 ± 3.3	113.3 ± 12.0	—	—	—
GCS, median (IQR)	15 (15–15)	15 (14–15)	15 (15–15)	15 (15–15)	Kruskal-Wallis	χ²=7.41	0.060
BMI (kg/m²), mean ± SD	27.9 ± 6.8	26.0 ± 5.6	25.7 ± 6.0	25.0 ± 5.3	ANOVA	F=7.42	<0.001
LOS (days), median (IQR)	1 (1–2)	1.5 (1–3)	3 (1–6)	4 (2–8)	Kruskal-Wallis	χ²=78.08	<0.001
Anticoagulation use, n (%)	42 (24.4%)	57 (33.1%)	76 (44.2%)	103 (60.2%)	Chi-square	χ²=28.28	<0.001
Mortality, n (%)	1 (0.58%)	1 (0.58%)	3 (1.74%)	9 (5.26%)	Fisher exact	—	0.056

Univariate analysis

Female patients demonstrated significantly higher mean GTOS values than male patients (93.8 ± 15.2 vs. 90.2 ± 15.8; Welch t=3.03, p=0.003). Patients on anticoagulation therapy had significantly lower mean GTOS values than those not on anticoagulation therapy (88.4 ± 14.8 vs. 94.8 ± 15.6; Welch t=5.54, p<0.001). GTOS differed significantly across injury diagnostic categories (ANOVA F=29.76, p<0.001). Mechanism of injury also demonstrated a significant relationship with GTOS (ANOVA F=3.40, p=0.005). Table 6 displays the results of these univariate analyses. 

**Table 6:** Biometric univariate analyses for GTOS GTOS, Geriatric Trauma Outcome Score; SD, standard deviation; ANOVA, analysis of variance.

Variable	GTOS, mean ± SD	Statistical test	Test statistic	p-value
Female sex	93.8 ± 15.2	Welch t-test	t=3.03	0.003
Male sex	90.2 ± 15.8	Welch t-test	—	—
Anticoagulation: Yes	88.4 ± 14.8	Welch t-test	t=5.54	<0.001
Anticoagulation: No	94.8 ± 15.6	Welch t-test	—	—
Injury diagnosis	—	ANOVA	F=29.76	<0.001
Mechanism of injury	—	ANOVA	F=3.40	0.005

Mean GTOS values were highest among patients with long bone fractures (103.4 ± 11.3) and lowest among patients with soft tissue injuries (88.4 ± 14.4) and other or unknown injuries (85.9 ± 12.8). Patients in motor vehicle collisions had higher mean GTOS values (104.3 ± 26.4) than falls (92.2 ± 15.0), despite falls presenting as the most frequent mechanism of injury. Post-hoc Tukey testing confirmed the observed differences in GTOS across injury diagnosis (ANOVA F=29.76, p<0.001) and mechanism of injury (ANOVA F=3.40, p=0.005). Mean GTOS measures for injury diagnosis and mechanism of injury are displayed in Table 7 and Table 8, respectively. 

**Table 7:** Mean GTOS by diagnosis of the injury TBI, traumatic brain injury; GTOS, Geriatric Trauma Outcome Score; SD, standard deviation.

Diagnosis	n (%)	GTOS, mean ± SD
TBIs and head injuries	249 (36.2%)	90.2 ± 15.9
Soft tissue injuries	196 (28.5%)	88.4 ± 14.4
Long bone fractures	113 (16.4%)	103.4 ± 11.3
Other fractures	52 (7.6%)	100.6 ± 15.4
Other or unknown injuries	77 (11.2%)	85.9 ± 12.8

**Table 8:** Mean GTOS by the mechanism of the injury GTOS, Geriatric Trauma Outcome Score; SD, standard deviation.

Mechanism	n (%)	GTOS, mean ± SD
Falls	616 (89.7%)	92.2 ± 15.0
Motor vehicle collisions	15 (2.2%)	104.3 ± 26.4
Pedestrians struck	10 (1.5%)	97.8 ± 27.8
Assaults	10 (1.5%)	84.2 ± 12.5
Bicycle accidents	8 (1.2%)	88.6 ± 18.9
Other or unknown mechanisms	28 (4.1%)	86.9 ± 11.6

**Figure 2:**
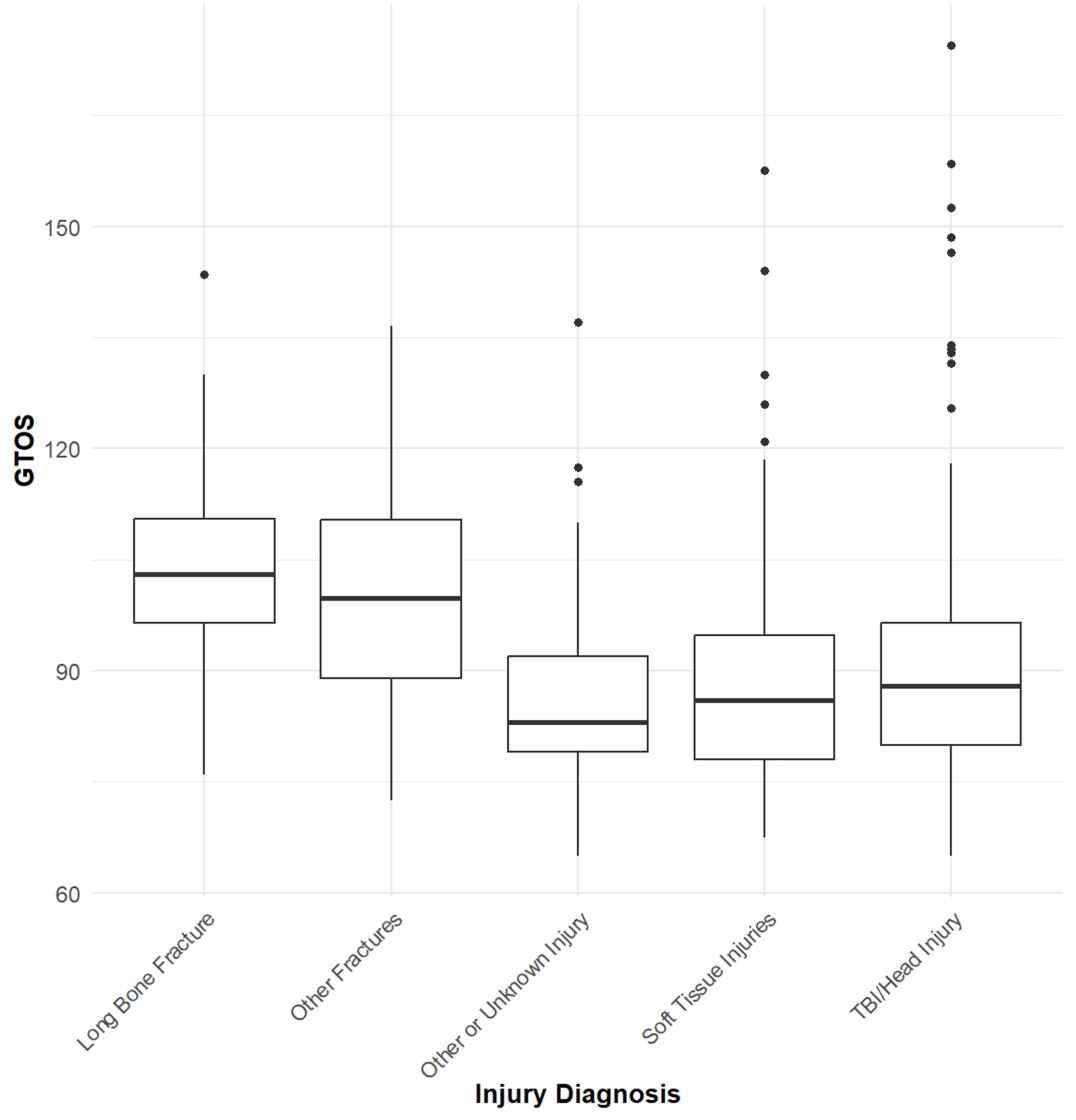
GTOS distribution by the diagnosis of the injury GTOS, Geriatric Trauma Outcome Score; TBI, traumatic brain injury.

**Figure 3:**
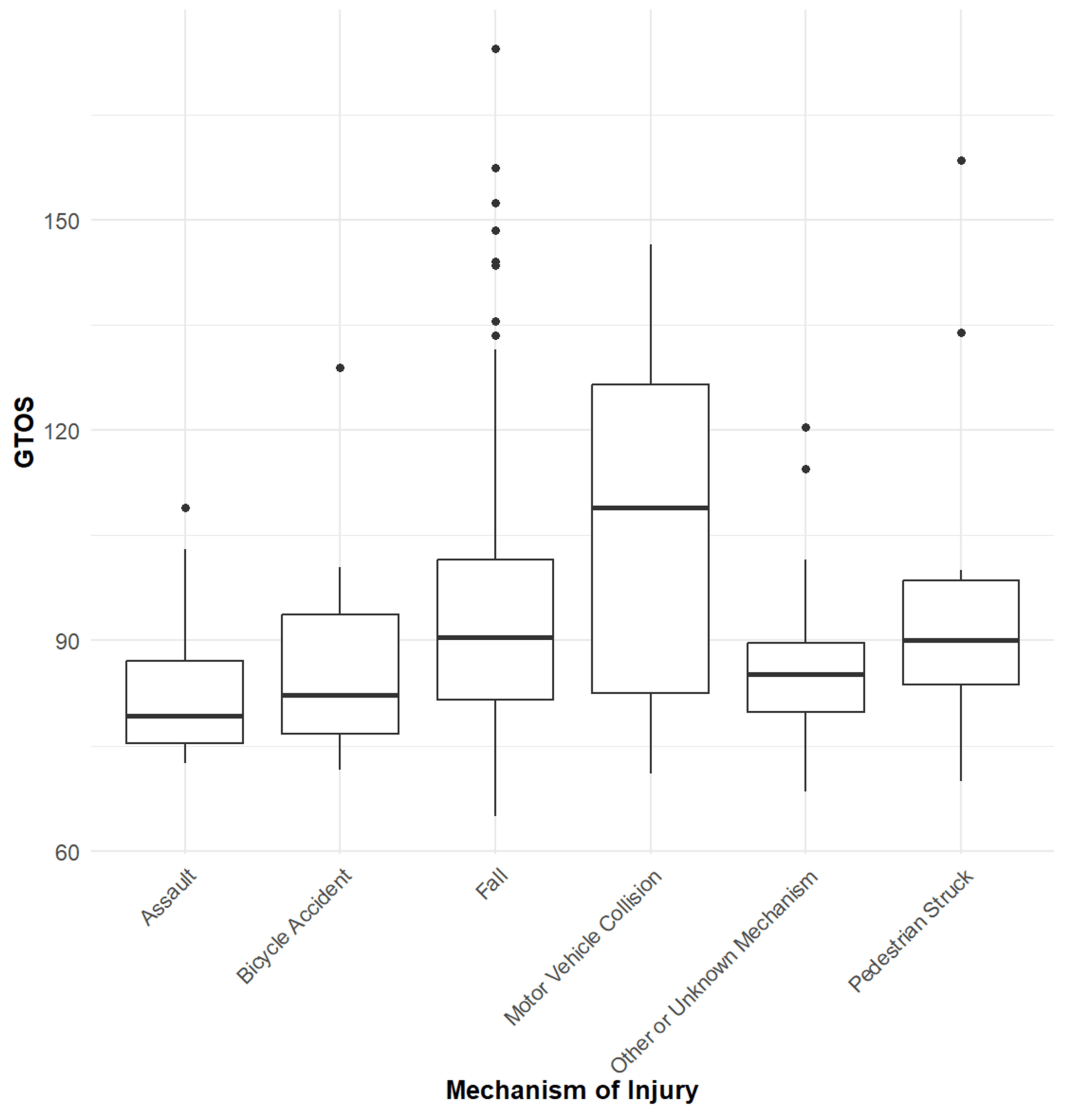
GTOS distribution by the mechanism of injury GTOS, Geriatric Trauma Outcome Score.

Table 7 and Table 8 can be visualized as interquartile plots, as observed in Figure 2 and Figure 3, respectively. 

GTOS maintained a weak inverse relationship with BMI (*ρ*=-0.18, p<0.001) and a moderate positive association with LOS (*ρ*=0.34, p<0.001). No significant association was observed between GTOS and GCS (*ρ*=-0.01, p=0.870). BMI and LOS demonstrated a weak inverse relationship (*ρ*=-0.09, p=0.010), as shown in Table 9.

**Table 9:** Spearman's ρ table GTOS, Geriatric Trauma Outcome Score; BMI, body mass index; GCS, Glasgow Coma Score; LOS, length of stay.

Variables	Spearman's *ρ*	p-value
GTOS vs. BMI	-0.18	<0.001
GTOS vs. GCS	-0.01	0.870
GTOS vs. LOS	0.34	<0.001
BMI vs. LOS	-0.09	0.010

Multivariate analysis

In our multivariable linear regression, lower BMI (β=-0.25, robust SE=0.08, p=0.002) and anticoagulation use (β=-3.57, robust SE=1.12, p=0.001) independently predicted lower GTOS values. Motor vehicle collisions were associated with significantly higher GTOS values (β=16.61, robust SE=7.44, p=0.026) relative to assaults, which served as the reference category. GCS demonstrated a nonsignificant inverse relationship with GTOS (β=-1.52, robust SE=0.99, p=0.126). Compared with long bone fractures, other or unknown injuries, soft tissue injuries, and TBIs and head injuries were independently associated with lower GTOS values. Other fractures showed a nonsignificant trend toward lower GTOS values relative to long bone fractures (β=-3.48, robust SE=2.32, p=0.133).

The overall model presented moderate explanatory fit (R² =0.212, adjusted R² =0.197; F=13.95, p<0.001). Heteroskedasticity was detected in the linear model via a Breusch-Pagan test (BP=47.99, p<0.001), warranting the inclusion of heteroskedasticity-consistent (HC1) robust SEs. Generalized variance inflation factor (GVIF) analysis demonstrated no clinically significant multicollinearity, with all adjusted GVIF values below 1.06. The full results of the multivariable model are displayed in Table 10.

**Table 10:** Multivariable linear regression model for predicting GTOS GTOS, Geriatric Trauma Outcome Score; BMI, body mass index; GCS, Glasgow Coma Score; TBI, traumatic brain injury; SE, standard error.

Variable	β coefficient	Robust SE	t-statistic	p-value	Standardized β
Constant	129.35	15.53	8.33	<0.001	—
Male sex	-1.67	1.10	-1.51	0.131	-0.053
BMI	-0.25	0.08	-3.11	0.002	-0.097
GCS	-1.52	0.99	-1.53	0.126	-0.097
Anticoagulation use	-3.57	1.12	-3.20	0.001	-0.113
Bicycle accidents	-2.13	6.10	-0.35	0.727	-0.015
Falls	4.27	3.67	1.17	0.244	0.084
Motor vehicle collisions	16.61	7.44	2.23	0.026	0.156
Other or unknown mechanisms	-0.59	3.91	-0.15	0.880	-0.008
Pedestrians struck	11.64	9.18	1.27	0.205	0.089
Other fractures	-3.48	2.32	-1.50	0.133	-0.060
Other or unknown injuries	-17.12	1.78	-9.62	<0.001	-0.345
Soft tissue injuries	-13.76	1.53	-8.97	<0.001	-0.399
TBIs and head injuries	-11.74	1.40	-8.36	<0.001	-0.362

Note that for the categorical predictor variables (i.e., male sex), the reference categories are female sex, no anticoagulation use, assaults (mechanism of injury), and long bone fractures (injury diagnosis). Standardized β coefficients are also reported for each predictor variable.

A parsimonious regression model was also used to predict GTOS. The parsimonious model tested included GCS, anticoagulation use, and injury diagnosis, which remained statistically significant overall (R² =0.179, adjusted R² =0.172; F=24.78, p<0.001). Use of HC1 robust SEs preserved significant associations between GTOS and lower GCS, anticoagulation use, and several injury diagnostic categories, including soft tissue injuries. Table 11 displays the results of this parsimonious model.

**Table 11:** Parsimonious regression model for predicting GTOS GTOS, Geriatric Trauma Outcome Score; GCS, Glasgow Coma Score; TBI, traumatic brain injury; SE, standard error.

Variable	β coefficient	Robust SE	t-statistic	p-value	Standardized β
Constant	132.13	14.13	9.35	<0.001	—
GCS	-1.88	0.96	-1.96	0.050	-0.120
Anticoagulation use	-4.06	1.13	-3.60	<0.001	-0.128
Other fractures	-3.16	2.35	-1.34	0.180	-0.054
Other or unknown injuries	-16.86	1.81	-9.32	<0.001	-0.341
Soft tissue injuries	-14.74	1.51	-9.76	<0.001	-0.427
TBIs and head injuries	-12.19	1.46	-8.35	<0.001	-0.376

Both the multivariable regression model and the parsimonious regression model generated stable estimates with similar magnitudes. Anticoagulation therapy and several injury diagnostic categories, including soft tissue injuries, TBIs, head injuries, and other or unknown injuries, remained significantly associated with lower GTOS values between models. Although the parsimonious model presented a slightly weaker overall fit (adjusted R² =0.172 vs. 0.197 for the original multivariable model), both the direction and coefficient sizes of the principal relationships remained within range of the multivariable model. Mechanisms of injury and other covariates excluded from the parsimonious model (e.g., falls) did not substantially alter the primary findings, although the parsimonious β coefficients were somewhat larger because fewer explanatory variables were included.

Firth penalized logistic regression

Exploratory modeling of mortality events was performed using the Firth penalized logistic regression to reduce sparse-event bias (n=14). Higher GTOS was associated with increased odds of mortality (OR=1.043, 95% CI: 1.015-1.070; p=0.003), while higher GCS had a significant relationship with reduced odds of mortality (OR=0.772, 95% CI: 0.623-0.980; p=0.036). The overall model was statistically significant (likelihood ratio χ² = 18.31, p<0.001). The results are presented in Table 12. 

**Table 12:** Firth penalized logistic regression for mortality events GTOS, Geriatric Trauma Outcome Score; GCS, Glasgow Coma Score; CI, confidence interval.

Variable	Odds Ratio	95% CI	Wald χ²	p-value
GTOS	1.043	1.015–1.070	9.09	0.003
GCS	0.772	0.623–0.980	4.41	0.036

## Discussion

In this retrospective cohort analysis of 687 geriatric trauma patients, GTOS had significant associations with several demographic and clinical variables and displayed meaningful stratification across injury diagnoses and mechanisms. Higher GTOS values were observed among patients with lower BMI and among those with specific injury mechanisms and diagnoses, including motor vehicle collisions and long bone fractures. Several of these relationships persisted after multivariable adjustment. This finding suggests that GTOS may reflect broader clinical and injury-related patterns beyond its original structural components. 

These findings are consistent with prior work supporting GTOS as a practical clinical tool within geriatric trauma populations. GTOS incorporates age, injury severity, and transfusion status, variables already well established in the trauma literature as contributors to patient prognosis and overall injury burden. In the retrospective cohort, higher GTOS values were most frequently observed among patients with long bone fractures and motor vehicle collisions. In contrast, lower values were observed among patients with soft tissue injuries, TBIs, head injuries, and other or unknown injuries. These findings likely reflect underlying differences in injury severity and trauma mechanisms across diagnostic categories.

Among mechanisms of injury, motor vehicle collisions had significantly higher GTOS values than the reference category and remained independently associated with GTOS after adjustment in a multivariable model. In contrast, falls, though accounting for the overwhelming majority of injuries in the retrospective cohort, were not independently associated with elevated GTOS values after controlling for additional covariates. This likely reflects the heterogeneity of fall-related injuries in geriatric trauma populations, ranging from relatively minor soft tissue injuries to more severe fractures such as spinal and hip fractures.

The primary multivariable regression model presented moderate explanatory power (adjusted R² =0.197), with findings remaining largely stable following the inclusion of robust SEs. Anticoagulation use and lower BMI independently predicted lower GTOS values, while several injury diagnostic categories showed persistent negative relationships relative to long bone fractures. GVIF analysis demonstrated no clinically meaningful multicollinearity, despite the inclusion of several related injury variables. Collectively, these findings support the internal stability of the overall regression structure.

The parsimonious regression model generated findings consistent with the primary multivariable analysis. Anticoagulation use and several injury diagnostic categories, including soft tissue injuries, TBIs, head injuries, and other or unknown injuries, remained significantly associated with GTOS values following model truncation. Although overall fit decreased modestly in the parsimonious model, the direction and relative magnitude of the primary relationships remained stable. This finding supports the robustness of the observed associations. Mechanisms of injury and additional covariates excluded from the parsimonious model did not substantially alter the primary findings. Across both models, anticoagulation use was significantly associated with lower GTOS values. 

Exploratory mortality modeling using the Firth penalized logistic regression showed that higher GTOS values were associated with increased odds of in-hospital mortality, whereas higher GCS scores were associated with lower mortality risk. The Firth penalized regression was used because only 14 mortality events occurred within the cohort. Conventional logistic regression may have produced sparse-event bias and unstable parameter estimates. Although these mortality analyses should be interpreted cautiously, the findings nevertheless support the potential relevance of GTOS within broader geriatric trauma risk assessment.

Stratification by GTOS quartiles further supported the clinical relevance of GTOS in care management. Increasing GTOS quartiles were associated with greater hospitalization burden, including progressively longer hospital LOS, higher anticoagulation prevalence, and increasing mortality frequency. Although mortality differences across quartiles did not reach statistical significance, likely because of the low overall mortality rate, the observed stepwise trend suggests clinically meaningful risk separation across increasing GTOS levels.

Several limitations warrant consideration. First, the relatively low number of mortality events limits overall statistical power for mortality-focused analyses and may reduce the precision of certain subgroup comparisons. Although Firth penalized logistic regression was employed specifically to mitigate bias resulting from low event frequency, this nevertheless warrants cautious interpretation of the study's exploratory mortality findings. Second, as with other retrospective studies, the use of retrospective data introduces the possibility of selection bias and confounding variable influence. Third, restriction to a single Level 1 trauma center may limit generalizability to other institutions or trauma systems. Finally, several mechanism and diagnostic categories contained relatively small sample sizes, which may contribute to wider CIs and thus less reliable coefficient estimations.

Despite these limitations, this study possesses several notable strengths. This study included a well-defined retrospective geriatric trauma cohort with detailed clinical characteristics. It also applied a comprehensive statistical framework, including multivariable regression modeling, robust SE estimation, quartile-based stratification, post-hoc Tukey testing, and exploratory Firth penalized logistic regression. Additionally, the inclusion of diagnosis-specific and mechanism-specific analyses enhances the clinical interpretability of GTOS and provides additional context regarding its potential application within geriatric trauma assessment. For example, patients with higher GTOS values may be at elevated mortality risk and thus require more intensive monitoring and heightened care interventions such as surgical standbys. Additionally, geriatric trauma patients involved in motor vehicle collisions demonstrated higher GTOS values. Given the observed relationship between GTOS and mortality, these patients may warrant particular clinical attention and monitoring. 

## Conclusions

The GTOS demonstrated meaningful relationships with several demographic, clinical, and injury-related characteristics in geriatric trauma patients, including injury diagnosis, mechanism of injury, anticoagulation use, and hospital LOS. Higher GTOS values were additionally associated with increased odds of in-hospital mortality through exploratory Firth penalized logistic regression analysis. Although the overall explanatory capacity was moderate, the findings remained generally stable across both multivariable and parsimonious regression frameworks. These results support the continued clinical utility of GTOS within geriatric trauma assessment while emphasizing the importance of integrating the score with additional clinical and injury-specific factors to improve overall risk stratification for the patients.
